# Trends in Blood Pressure and Hypertension Among US Children and Adolescents, 1999-2018

**DOI:** 10.1001/jamanetworkopen.2021.3917

**Published:** 2021-04-01

**Authors:** Shakia T. Hardy, Swati Sakhuja, Byron C. Jaeger, Elaine M. Urbina, Shakira F. Suglia, Daniel I. Feig, Paul Muntner

**Affiliations:** 1 Department of Epidemiology, University of Alabama at Birmingham; 2 Department of Biostatistics, University of Alabama at Birmingham; 3 The Heart Institute, Cincinnati Children’s Hospital, University of Cincinnati, Cincinnati, Ohio; 4 Department of Epidemiology, Emory University, Atlanta, Georgia; 5 Division of Pediatric Nephrology, University of Alabama at Birmingham

## Abstract

**Importance:**

Higher blood pressure (BP) levels in children are associated with an increased risk for hypertension and subclinical cardiovascular disease in adulthood. Identifying trends in BP could inform the need for interventions to lower BP.

**Objective:**

To determine whether systolic BP (SBP) and diastolic BP (DBP) levels among US children have changed during the past 20 years.

**Design, Setting, and Participants:**

This serial cross-sectional analysis of National Health and Nutrition Examination Survey data included 9117 children aged 8 to 12 years and 10 156 adolescents aged 13 to 17 years, weighted to the US population from 1999-2002 to 2015-2018. Data were collected from March 1999 to December 2018 and analyzed from March 26, 2020, to February 2, 2021.

**Exposures:**

Calendar year.

**Main Outcomes and Measures:**

The primary outcomes were mean SBP and mean DBP.

**Results:**

A total of 19 273 participants were included in the analysis. Among children aged 8 to 12 years in 2015-2018 (mean age, 10.5 [95% CI, 10.5-10.6] years), 48.7% (95% CI, 45.2%-52.2%) were girls and 51.3% (95% CI, 47.8%-54.8%) were boys; 49.7% (95% CI, 42.2%-57.1%) were non-Hispanic White; 13.7% (95% CI, 10.3%-18.1%) were non-Hispanic Black; 25.5% (95% CI, 19.9%-32.0%) were Hispanic; 4.7% (95% CI, 3.2%-6.7%) were non-Hispanic Asian; and 6.5% (95% CI, 4.9%-8.5%) were other non-Hispanic race/ethnicity. Among those aged 13 to 17 years in 2015-2018 (mean age, 15.5 [95% CI, 15.5-15.5] years), 49.1% (95% CI, 46.1%-52.2%) were girls and 50.9% (95% CI, 47.8%-53.9%) were boys; 53.3% (95% CI, 46.4%-60.1%) were non-Hispanic White; 13.9% (95% CI, 10.3%-18.7%) were non-Hispanic Black; 21.9% (95% CI, 16.6%-28.2%) were Hispanic; 4.6% (95% CI, 3.2%-6.5%) were non-Hispanic Asian; and 6.3% (95% CI, 4.7%-8.5%) were other non-Hispanic race/ethnicity. Among children aged 8 to 12 years, age-adjusted mean SBP decreased from 102.4 (95% CI, 101.7-103.1) mm Hg in 1999-2002 to 101.5 (95% CI, 100.8-102.2) mm Hg in 2011-2014 and then increased to 102.5 (95% CI, 101.9-103.2) mm Hg in 2015-2018. Age-adjusted mean DBP decreased from 57.2 (95% CI, 56.5-58.0) mm Hg in 1999-2002 to 51.9 (95% CI, 50.1-53.7) mm Hg in 2011-2014 and increased to 53.2 (95% CI, 52.2-54.1) mm Hg in 2015-2018. Among adolescents aged 13 to 17 years, age-adjusted mean SBP decreased from 109.2 (95% CI, 108.7-109.7) mm Hg in 1999-2002 to 108.4 (95% CI, 107.8-109.1) mm Hg in 2011-2014 and remained unchanged in 2015-2018 (108.4 [95% CI, 107.8-109.1] mm Hg). Mean DBP decreased from 62.6 (95% CI, 61.7-63.5) mm Hg in 1999-2002 to 59.6 (95% CI, 58.2-60.9) mm Hg in 2011-2014 and then increased to 60.8 (95% CI, 59.8-61.7) mm Hg in 2015-2018. Among children aged 8 to 12 years, mean SBP was 3.2 (95% CI, 1.7-4.6) mm Hg higher among those with overweight and 6.8 (95% CI, 5.6-8.1) mm Hg higher among those with obesity compared with normal weight; mean DBP was 3.2 (95% CI, 0.7-5.6) mm Hg higher among those with overweight and 3.5 (95% CI, 1.9- 5.1) mm Hg higher among those with obesity compared with normal weight. Among adolescents aged 13 to 17 years, mean SBP was 3.5 (95% CI 1.9-5.1) mm Hg higher among those with overweight and 6.6 (95% CI, 5.2-8.0) mm Hg higher among those with obesity compared with normal weight, 4.8 (95% CI, 3.8-5.8) mm Hg higher among boys compared with girls, and 3.0 (95% CI, 1.7-4.3) mm Hg higher among non-Hispanic Black compared with non-Hispanic White participants.

**Conclusions and Relevance:**

Despite an overall decline in mean SBP and DBP from 1999-2002 to 2015-2018, BP levels among children and adolescents may have increased from 2011-2014 to 2015-2018.

## Key Points

**Question:**

Have systolic blood pressure (SBP) and diastolic blood pressure (DBP) levels among US children changed during the past 20 years?

**Findings:**

In this serial cross-sectional study of 19 273 children and adolescents included in the National Health and Nutrition Examination Survey (NHANES), age-adjusted mean SBP was lower in the 2015-2018 cycle compared with the 1999-2002 cycle among adolescents aged 13 to 17 years, and mean DBP was lower in the 2015-2018 cycle compared with the 1999-2002 cycle among children aged 8 to 12 and adolescents aged 13 to 17 years.

**Meaning:**

These representative findings suggest that from 1999-2002 to 2015-2018, mean SBP decreased among adolescents aged 13 to 17 years and mean DBP decreased among children and adolescents aged 8 to 12 and 13 to 17 years, respectively, in the US.

## Teaser

This cross-sectional study assesses data from the National Health and Nutrition Examination Survey to determine whether systolic and diastolic blood pressure levels among US children and adolescents have changed during the past 20 years.

## Introduction

Hypertension is one of the most important modifiable risk factors for morbidity and mortality due to cardiovascular disease (CVD).^1,2^ Longitudinal studies have shown that higher blood pressure (BP) levels in childhood are associated with an early onset of hypertension in adulthood.^3,4^ Furthermore, epidemiologic and pathophysiologic studies^5,6,7^ suggest that hypertension in childhood is associated with subclinical atherosclerosis, target organ damage, and increased CVD risk in adulthood, emphasizing the importance of identifying and preventing increases in BP levels and hypertension in childhood.

Studies have shown that the proportion of US children and adolescents with elevated BP and hypertension may have decreased from 2005-2008 to 2013-2016.^8,9,10^ However, a study among US adults reported that systolic BP (SBP) and diastolic BP (DBP) increased from 2013-2014 to 2017-2018.^11^ Whether the trend of increasing SBP and DBP among adults extends to children and adolescents remains unknown. Estimating 20-year trends in the distribution of SBP and DBP and the prevalence of elevated BP and hypertension with the 2017 American Academy of Pediatrics Clinical Practice Guideline definitions^12^ could identify differences in BP among subpopulations and inform the need for evidence-based interventions to prevent increases in BP among children who are at increased risk for hypertension.

We analyzed data from 10 cycles of the US National Health and Nutrition Examination Survey (NHANES) to determine whether reported decreases in the prevalence of elevated BP and hypertension from 2005-2008 to 2013-2016 among US children and adolescents have continued through 2017-2018 and whether these changes reflect shifts in the entire distributions of SBP and DBP. In addition, we determined whether mean SBP and DBP and elevated BP and hypertension prevalence were different in 2015-2018 between groups defined by demographic and socioeconomic factors and body mass index (BMI).

## Methods

NHANES is a series of cross-sectional studies that assess the health and nutritional status of the noninstitutionalized US population. Since the 1999-2000 cycle, the National Center for Health Statistics (NCHS) has conducted NHANES in 2-year cycles using a complex, multistage probability sampling design to select participants such that nationally representative estimates can be generated. Sociodemographic and cardiovascular health measures for this study were obtained from the publicly available data files for the 10 NHANES cycles conducted from 1999-2000 through 2017-2018. The NCHS institutional review board approved the study protocol for each NHANES cycle. Written informed consent was provided by parents for all participants younger than 18 years and by children and adolescents 12 years or older. Written assent was provided by children aged 8 to 11 years. This study adheres to the Strengthening the Reporting of Observational Studies in Epidemiology (STROBE) reporting guideline.

Each NHANES cycle included an in-home interview and physical examination conducted at a mobile examination center. Two-year NHANES cycles were pooled into 4-year groups to provide more stable estimates. We restricted the analysis to children and adolescents aged 8 to 17 years who completed both the NHANES interview and physical examination (n = 20 523). We excluded participants who did not have at least 1 SBP and DBP measurement (n = 1215). We also excluded children aged 8 to 12 years who were missing information on height because height is used to determine BP percentiles in this age group (n = 35). After these exclusions, a total of 19 273 participants (9117 children aged 8 to 12 years and 10 156 adolescents aged 13 to 17 years) were included in the analysis (eFigure in the Supplement).

### Data Collection

#### Sociodemographic Characteristics and Weight Status

Data for this study were collected from March 1999 to December 2018. Trained interviewers obtained information on sociodemographic and health characteristics of participants using standardized questionnaires. Age at the time of the NHANES examination was grouped into categories of 8 to 12 years and 13 to 17 years to align with BP category definitions in the 2017 American Academy of Pediatrics Clinical Practice Guideline.^12^ From the 1999-2002 through the 2007-2010 cycles, race/ethnicity categories in the publicly available NHANES data sets included non-Hispanic White, non-Hispanic Black, Hispanic, and other race/ethnicity. Beginning in the 2011-2012 cycle, the publicly available NHANES data sets included non-Hispanic Asian as its own category. The NCHS calculated the poverty-to-income ratio (PIR) as a ratio of self- or proxy-reported family income to the federal poverty level based on family size. A PIR of less than 1.00 indicates that the family income was below the poverty level. The PIR was grouped as less than 1.30, 1.30 to 3.49, and 3.50 or greater, consistent with categories frequently used by the NCHS.^13^

The NHANES examination included height and weight measurements performed by trained health technicians using a standardized protocol. Body mass index, calculated as weight in kilograms divided by height in meters squared and rounded to 1 decimal place, was categorized as normal (5th percentile to <85th percentile), overweight (85th percentile to <95th percentile), and obesity (≥95th percentile) based on age- and sex-specific growth charts developed by the Centers for Disease Control and Prevention in 2000.^14^ Due to the small sample size, children with a BMI of less than the 5th percentile are not presented.

#### BP Measurement and Antihypertensive Use

Blood pressure was measured during the examination using the same standardized protocol for each NHANES cycle. Trained physicians measured SBP and DBP using a mercury sphygmomanometer and an appropriately sized cuff. After 5 minutes of seated rest, 3 BP measurements were obtained at 30-second intervals. The mean of all available measurements was used to define SBP and DBP for each participant. Among participants included in the current analysis, 1012 (5.3%) had 1, 1226 (6.4%) had 2, and 17 035 (88.4%) had 3 SBP measurements, and 1240 (6.4%) had 1, 1569 (8.1%) had 2, and 16 464 (85.4%) had 3 DBP measurements. Physicians were certified annually through a BP measurement training program and recertified quarterly if needed to ensure quality of the BP measurements.

#### Definitions of Hypertension and Elevated BP

The 2017 American Academy of Pediatrics Clinical Practice Guideline was used to define normal BP, elevated BP, and hypertension.^12^ Among children aged 8 to 12 years, age-, sex-, and height-specific SBP and DBP percentile tables were used for defining BP categories.^12^ Normal BP was defined as SBP and DBP of less than the 90th percentile; elevated BP, as SBP and/or DBP from the 90th to less than the 95th percentile or SBP of at least 120 mm Hg to less than the 95th percentile and DBP of less than 80 mm Hg to less than the 95th percentile; and hypertension, as SBP and/or DBP of at least the 95th percentile or greater or SBP of at least 130 mm Hg and/or DBP of at least 80 mm Hg (eTable 1 in the Supplement). For adolescents aged 13 to 17 years, normal BP was defined as SBP of less than 120 mm Hg and DBP of less than 80 mm Hg; elevated BP, as SBP of 120 to 129 mm Hg and DBP of less than 80 mm Hg; and hypertension, as SBP of at least 130 mm Hg and/or DBP of at least 80 mm Hg. High BP for children aged 8 to 12 or adolescents aged 13 to 17 years was defined by having elevated BP or hypertension.

### Statistical Analysis

Data were analyzed from March 26, 2020, to February 2, 2021. All analyses were performed for children aged 8 to 12 years and adolescents aged 13 to 17 years separately. Characteristics of US children and adolescents were calculated for each 4-year NHANES cycle: 1999-2002, 2003-2006, 2007-2010, 2011-2014, and 2015-2018. We calculated age-adjusted mean SBP and DBP, SBP and DBP percentiles (5th, 15th, 25th, 50th, 75th, 85th, and 95th), and the prevalence of normal BP, elevated BP, and hypertension. Estimates are provided for the overall population and within a priori selected subgroups defined by sex, race/ethnicity, BMI, and PIR. Linear regression was used to assess linear trends in mean SBP, mean DBP, and BP percentiles, and logistic regression was used to assess trends in elevated BP and hypertension prevalence across the 4-year cycles. We tested for multiplicative interactions between each covariate (sex, race/ethnicity, BMI, and PIR) and survey year. These tests were replicated using each outcome, mean SBP and DBP, and prevalence of normal BP, elevated BP, and hypertension as the dependent variable. Statistical significance was defined by a 2-sided *P* < .05.

Using NHANES data from 2015-2018, we determined factors associated with mean SBP, mean DBP, hypertension, and high BP. We used linear regression to estimate differences in mean SBP and DBP and Poisson regression to estimate prevalence ratios (PRs) for hypertension and high BP associated with age, sex, race/ethnicity, BMI, and PIR. Models were conducted with adjustment for age, sex, and race/ethnicity and all factors simultaneously.

NHANES sampling weights were used in all calculations to obtain US nationally representative estimates. Age adjustment was performed using direct standardization with the age distribution of children aged 8 to 12 years and adolescents aged 13 to 17 years from NHANES 1999 to 2018 used as the standard population. The standard population distribution was 19.2% for those aged 8 years; 20.1%, aged 9 years; 20.1%, aged 10 years; 20.0%, aged 11 years; and 20.6%, aged 12 years. The standard population distribution was 19.4% for those aged 13 years; 21.1%, aged 14 years; 19.5%, aged 15 years; 21.0%, aged 16 years; and 18.9%, aged 17 years. Data management was conducted using SAS, version 9.4 (SAS Institute Inc). Data analysis was conducted using STATA, version 16 (StataCorp LLC) and R, version 4.0.1 (R Program for Statistical Computing).

## Results

In the 1999-2002 and 2015-2018 cycles, 47.7% (95% CI, 45.1%-50.3%) and 48.7% (95% CI, 45.2%-52.2%) of US children aged 8 to 12 years, respectively, were girls; 52.3% (95% CI, 49.7%-54.9%) and 51.3% (95% CI, 47.8%-54.8%), respectively, were boys; 59.1% (95% CI, 53.8%-64.2%) and 49.7% (95% CI, 42.2%-57.1%), respectively, were non-Hispanic White; 16.3% (95% CI, 12.7%-20.6%) and 13.7% (95% CI, 10.3%-18.1%), respectively, were non-Hispanic Black; 19.8% (95% CI, 15.4%-25.1%) and 25.5% (95% CI, 19.9%-32.0%), respectively, were Hispanic; and 4.8% (95% CI, 3.6%-6.3%) and 6.5% (95% CI, 4.9%-8.5%), respectively, were of other non-Hispanic race/ethnicity (Table 1). Mean age ranged from 10.5 (95% CI, 10.5-10.6) years in 1999-2002 to 10.5 (95% CI, 10.5-10.5) years in 2015-2018. In the 1999-2002 and 2015-2018 cycles, 50.2% (95% CI, 47.4%-53.0%) and 49.1% (95% CI, 46.1%-52.2%), respectively, of US adolescents aged 13 to 17 years were girls; 49.8% (95% CI, 47.0%-52.6%) and 50.9% (95% CI, 47.8%-53.9%), respectively, were boys; 60.1% (95% CI, 56.1%-64.0%) and 53.3% (95% CI, 46.4%-60.1%), respectively, were non-Hispanic White; 14.1% (95% CI, 11.0%-17.8%) and 13.9% (95% CI, 10.3%-18.7%), respectively, were non-Hispanic Black; 18.1% (95% CI, 14.1%-23.1%) and 21.9% (95% CI, 16.6%-28.2%), respectively, were Hispanic; and 7.7% (95% CI, 5.7%-10.3%) and 6.3% (95% CI, 4.7%-8.5%), respectively, were other non-Hispanic race/ethnicity. Mean age was 15.5 (95% CI, 15.5-15.5) throughout the NHANES cycles. In 2015-2018, 4.7% (95% CI, 3.2%-6.7%) of children aged 8 to 12 years and 4.6% (95% CI, 3.2%-6.5%) of adolescents aged 13 to 17 years were non-Hispanic Asian.

**Table 1.** Characteristics of US Children and Adolescents From 1999-2002 to 2015-2018

Study participant characteristic	NHANES cycle^a^					*P* value
	1999-2002	2003-2006	2007-2010	2011-2014	2015-2018	
**Children aged 8-12 y**						
NHANES sample size, No.	1981	1805	1808	1854	1669	NA
Age, mean (95% CI), y	10.5 (10.5-10.6)	10.5 (10.5-10.6)	10.5 (10.5-10.6)	10.5 (10.5-10.6)	10.5 (10.5-10.5)	.44
Sex						
Female	47.7 (45.1-50.3)	48.1 (44.7-51.9)	49.8 (47.0-52.6)	49.3 (46.0-52.7)	48.7 (45.2-52.2)	.53
Male	52.3 (49.7-54.9)	51.7 (48.1-55.3)	50.2 (47.4-53.0)	50.7 (47.3-54.0)	51.3 (47.8-54.8)	
Race/ethnicity						
Non-Hispanic White	59.1 (53.8-64.2)	57.6 (51.0-63.9)	56.7 (50.6-62.5)	54.7 (47.8-61.4)	49.7 (42.2-57.1)	.002
Non-Hispanic Black	16.3 (12.7-20.6)	15.9 (12.7-19.8)	13.8 (11.3-16.8)	13.5 (10.7-16.8)	13.7 (10.3-18.1)	
Hispanic	19.8 (15.4-25.1)	18.1 (14.4-22.6)	21.5 (16.8-27.0)	23.1 (17.9-29.3)	25.5 (19.9-32.0)	
Non-Hispanic Asian^b^	NA	NA	NA	4.3 (3.3-5.4)	4.7 (3.2-6.7)	
Non-Hispanic other	4.8 (3.6-6.3)	8.4 (6.0-11.7)	8.0 (6.2-10.4)	4.5 (3.4-5.9)	6.5 (4.9-8.5)	
BMI^c^						
Normal	65.6 (61.9-69.1)	62.5 (57.9-66.9)	60.5 (58.3-62.6)	61.9 (58.2-65.5)	61.6 (57.7-65.4)	.12
Overweight	16.8 (14.6-19.2)	18.1 (15.7-20.8)	18.1 (15.7-20.8)	17.6 (15.3-20.0)	17.5 (15.5-19.6)	
Obesity	17.6 (14.7-20.9)	19.4 (16.4-22.8)	21.5 (19.7-23.4)	20.5 (18.0-23.4)	20.9 (18.1-24.1)	
PIR^d^						
<1.30	35.2 (31.4-39.2)	29.5 (25.3-34.0)	33.0 (28.7-37.7)	36.3 (30.9-42.0)	30.0 (26.2-34.2)	.73
1.30-3.49	37.1 (33.0-41.4)	40.0 (35.8-44.3)	35.4 (31.8-39.2)	34.3 (30.3-38.5)	41.2 (37.3-45.3)	
≥3.50	27.7 (24.1-31.7)	30.5 (25.1-36.6)	31.6 (27.2-36.4)	29.5 (24.2-35.3)	28.7 (28.7-33.7)	
**Adolescents aged 13-17 y**						
NHANES sample size, No.	2889	2672	1522	1588	1485	NA
Age, mean (95% CI), y	15.5 (15.5-15.5)	15.5 (15.5-15.5)	15.5 (15.5-15.5)	15.5 (15.5-15.5)	15.5 (15.5-15.5)	.61
Sex						
Female	50.2 (47.4-53.0)	50.0 (47.5-52.6)	48.9 (45.8-52.1)	50.7 (47.2-54.3)	49.1 (46.1-52.2)	.74
Male	49.8 (47.0-52.6)	50.0 (47.4-52.5)	51.1 (47.9-54.2)	49.3 (45.7-52.8)	50.9 (47.8-53.9)	
Race/ethnicity						
Non-Hispanic White	60.1 (56.1-64.0)	64.5 (58.9-69.8)	59.8 (54.4-64.9)	54.0 (46.9-61.0)	53.3 (46.4-60.1)	.004
Non-Hispanic Black	14.1 (11.0-17.8)	14.5 (11.5-18.2)	14.9 (12.5-17.7)	15.0 (11.2-19.7)	13.9 (10.3-18.7)	
Hispanic	18.1 (14.1-23.1)	15.5 (12.4-19.2)	18.4 (14.2-23.4)	22.0 (17.4-27.5)	21.9 (16.6-28.2)	
Non-Hispanic Asian^b^	NA	NA	NA	5.3 (4.4-6.4)	4.6 (3.2-6.5)	
Non-Hispanic other	7.7 (5.7-10.3)	5.4 (3.8-7.7)	7.0 (5.1-9.4)	3.7 (2.6-5.3)	6.3 (4.7-8.5)	
BMI^c^						
Normal	68.5 (66.4-70.4)	65.3 (61.5-68.9)	65.0 (62.0-68.0)	63.1 (59.6-66.4)	59.2 (56.4-61.9)	<.001
Overweight	15.1 (13.5-16.8)	17.2 (14.9-19.9)	15.8 (13.8-18.2)	15.3 (13.2-17.6)	19.2 (17.5-21.0)	
Obesity	16.5 (15.2-17.8)	17.4 (14.9-20.3)	19.1 (16.3-22.3)	21.6 (18.6-25.0)	21.6 (18.8-24.6)	
PIR^d^						
<1.30	30.5 (27.6-33.6)	24.8 (21.8-28.1)	29.1 (25.6-32.7)	30.6 (25.3-36.4)	26.6 (22.3-31.4)	.42
1.30-3.49	36.2 (32.5-40.2)	38.1 (34.5-41.8)	35.3 (30.8-40.1)	38.6 (34.6-42.8)	43.0 (39.1-46.9)	
≥3.50	33.2 (29.6-37.1)	37.1 (32.6-41.9)	35.6 (30.3-41.3)	30.8 (25.6-36.7)	30.4 (25.5-35.8)	

Abbreviations: BMI, body mass index (calculated as weight in kilograms divided by height in meters squared); NA, not applicable; NHANES, National Health and Nutrition Examination Survey; PIR, poverty-to-income ratio.

^a^ Unless otherwise indicated, data are expressed as percentage (95% CI).

^b^ Non-Hispanic Asian participants were not categorized in 1999-2002, 2003-2006, and 2007-2010 owing to small sample sizes.

^c^ Normal indicates BMI of 5th percentile to less than 85th percentile; overweight, 85th percentile to less than 95th percentile; and obesity, 95th percentile or greater. Categories were based on age- and sex-specific growth charts developed by the Centers for Disease Control and Prevention in 2000. Owing to a small sample size, children with a BMI less than the 5th percentile are not presented.

^d^ A PIR <1.30 indicates that the family income was below 130% of the poverty level.

### Trends in the Distribution of SBP and DBP

**Figure 1.**
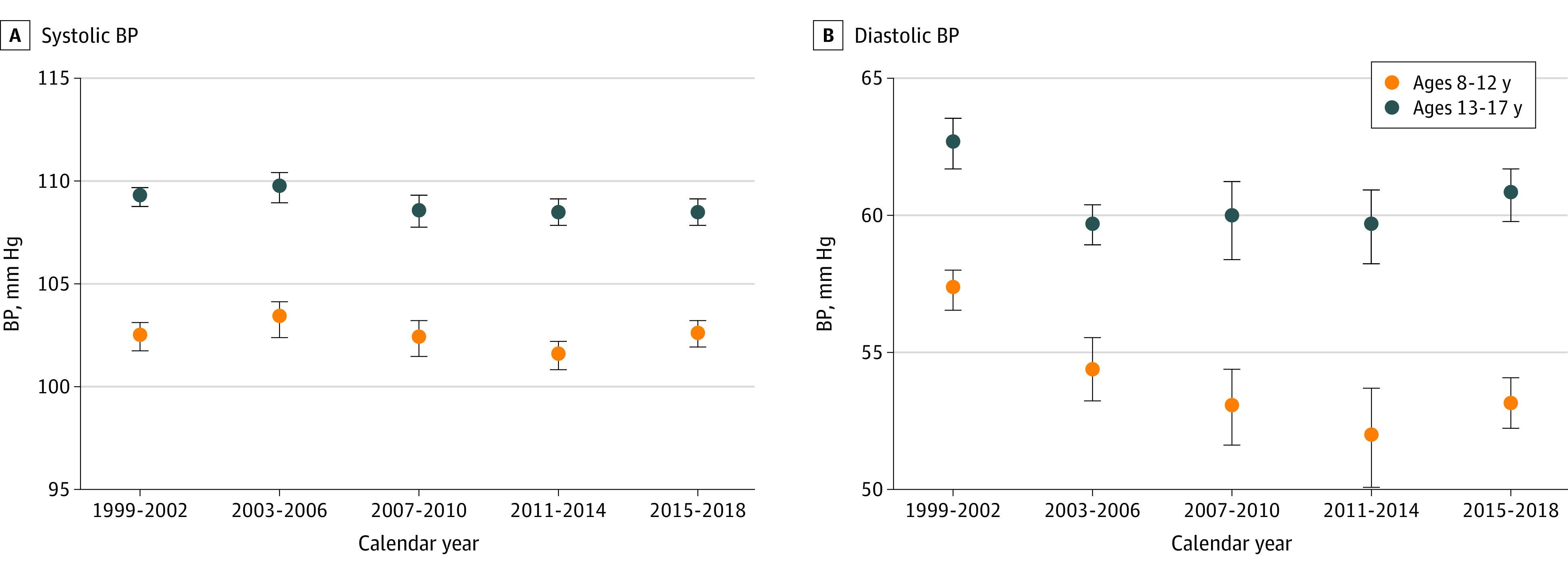
Age-Adjusted Mean Systolic and Diastolic Blood Pressure (BP) Among US Children and Adolescents Children were aged 8 to 12 years; adolescents, 13 to 17 years. Data are from the 1999-2002 to 2015-2018 cycles of the National Health and Nutrition Examination Survey. Age adjustment was performed using direct standardization, with the standard being US children and adolescents across the entire period from 1999 to 2018. Error bars represent 95% CIs.

Among children aged 8 to 12 years, the age-adjusted mean SBP decreased from 102.4 (95% CI, 101.7-103.1) mm Hg in 1999-2002 to 101.5 (95% CI, 100.8-102.2) mm Hg in 2011-2014 and then increased to 102.5 (95% CI, 101.9-103.2) mm Hg in 2015-2018 (*P* = .21 for trend from 1999-2002 through 2015-2018) (Figure 1A and eTable 2 in the Supplement). Among adolescents aged 13 to 17 years, age-adjusted mean SBP decreased from 109.2 (95% CI, 108.7-109.7) mm Hg in 1999-2002 to 108.4 (95% CI, 107.8-109.1) mm Hg in 2011-2014 and remained unchanged (108.4 [95% CI, 107.8-109.1] mm Hg) in 2015-2018 (*P* = .007 for trend from 1999-2002 through 2015-2018).

Among children aged 8 to 12 years, age-adjusted mean DBP decreased from 57.2 (95% CI, 56.5-58.0) mm Hg in 1999-2002 to 51.9 (95% CI, 50.1-53.7) mm Hg in 2011-2014 and then increased to 53.2 (95% CI, 52.2-54.1) mm Hg in 2015-2018 (*P* < .001 for trend from 1999-2002 through 2015-2018) (Figure 1B). Among adolescents aged 13 to 17 years, mean DBP decreased from 62.6 (95% CI, 61.7-63.5) mm Hg in 1999-2002 to 59.6 (95% CI, 58.2-60.9) mm Hg in 2011-2014 and then increased to 60.8 (95% CI, 59.8-61.7) mm Hg in 2015-2018 (*P* = .03 for trend from 1999-2002 through 2015-2018). The SBP and DBP distributions (5th, 15th, 25th, 50th, 75th, 85th, and 95th percentiles) for those aged 8 to 12 and 13 to 17 years are shown in eTable 3 in the Supplement.

### Trends in Age-Adjusted Prevalence of Hypertension

**Figure 2.**
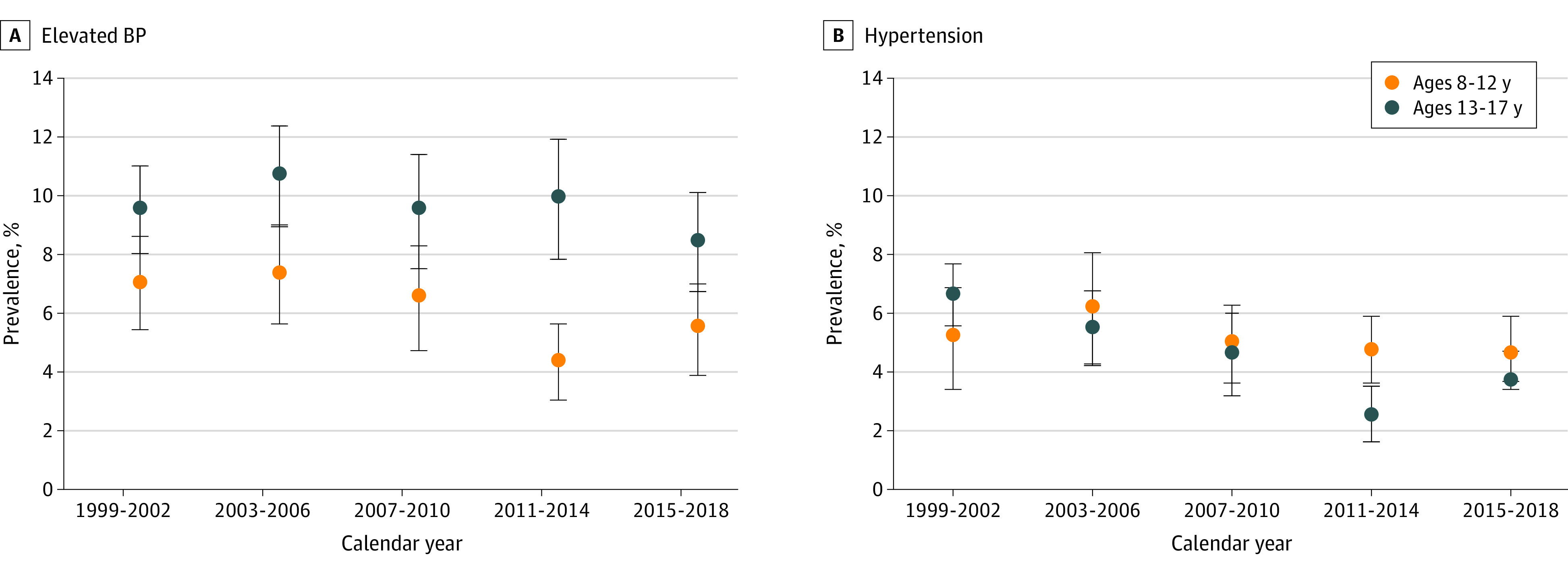
Age-Adjusted Prevalence of Elevated Blood Pressure (BP) and Hypertension Among US Children and Adolescents Data are from the 1999-2002 to 2015-2018 cycles in the National Health and Nutrition Examination Survey. Age adjustment was performed using direct standardization, with the standard being US children (aged 8-12 years) and adolescents (aged 13-17 years) across the entire period from 1999 to 2018. Error bars represent 95% CIs.

Among US children aged 8 to 12 years, the prevalence of hypertension increased from 5.2% (95% CI, 3.4%-6.9%) in 1999-2002 to 6.2% (95% CI, 4.3%-8.1%) in 2003-2006 and then decreased to 4.6% (95% CI, 3.4%-5.9%) in 2015-2018 (Figure 2B and eTable 4 in the Supplement) (*P* = .30 for trend from 1999-2002 to 2015-2018). Among adolescents aged 13 to 17 years, the age-adjusted prevalence of hypertension decreased from 6.6% (95% CI, 5.6%-7.7%) in 1999-2002 to 2.5% (95% CI, 1.6%-3.5%) in 2011-2014 and then increased to 3.7% (95% CI, 2.6%-4.7%) in 2015-2018 (*P* < .001 for trend from 1999-2002 to 2015-2018).

### Factors Associated With SBP and DBP Levels in NHANES 2015-2018

Among children aged 8 to 12 years, mean SBP was 3.2 (95% CI, 1.7-4.6) mm Hg higher among those with overweight and 6.8 (95% CI, 5.6-8.1) mm Hg higher among those with obesity compared with normal weight; mean DBP was 3.2 (95% CI, 0.7-5.6) mm Hg higher among those with overweight and 3.5 (95% CI, 1.9-5.1) mm Hg higher among those with obesity compared with normal weight (Table 2). Among adolescents aged 13 to 17 years, mean SBP was 3.5 (95% CI, 1.9-5.1) mm Hg higher among those with overweight and 6.6 (95% CI, 5.2 to 8.0) mm Hg higher among those with obesity compared with normal weight, 4.8 (95% CI, 3.8-5.8) mm Hg higher among boys compared with girls, and 3.0 (95% CI, 1.7-4.3) mm Hg higher among non-Hispanic Black compared with non-Hispanic White participants.

**Table 2.** Factors Associated With Systolic and Diastolic Blood Pressure Among US Children and Adolescents in 2015-2018

Study participant characteristic	Blood pressure, mm Hg							
	Systolic				Diastolic			
	Model 1^a^		Model 2^b^		Model 1^a^		Model 2^b^	
	Mean difference (95% CI)	*P* value	Mean difference (95% CI)	*P* value	Mean difference (95% CI)	*P* value	Mean difference (95% CI)	*P* value
**Children aged 8-12 y **								
Age, y								
8	0 [Reference]	NA	0 [Reference]	NA	0 [Reference]	NA	0 [Reference]	NA
9	2.3 (0.0 to 4.5)	.05	2.7 (0.7 to 4.7)	.01	2.6 (0.4 to 4.7)	.02	2.6 (0.3 to 4.9)	.03
10	2.8 (1.4 to 4.3)	<.001	2.9 (1.5 to 4.2)	<.001	3.0 (−0.4 to 6.5)	.08	3.0 (−0.7 to 6.7)	.11
11	4.6 (2.5 to 6.6)	<.001	4.7 (2.9 to 6.4)	<.001	5.1 (2.3 to 8.0)	.001	4.8 (1.5 to 8.0)	.005
12	6.0 (4.6 to 7.3)	<.001	6.1 (4.5 to 7.7)	<.001	8.9 (6.3 to 11.5)	<.001	9.2 (6.3 to 12.1)	<.001
Sex								
Female	0 [Reference]	NA	0 [Reference]	NA	0 [Reference]	NA	0 [Reference]	NA
Male	0.9 (0.1 to 1.8)	.03	1.0 (0.0 to 1.9)	.05	−1.5 (−3.3 to 0.3)	.10	−1.4 (−0.3 to 4.1)	.13
Race/ethnicity								
Non-Hispanic White	0 [Reference]	NA	0 [Reference]	NA	0 [Reference]	NA	0 [Reference]	NA
Non-Hispanic Black	1.0 (−0.2 to 2.2)	.11	−0.5 (−1.7 to 0.8)	.44	1.9 (−0.1 to 4.0)	.07	1.9 (−0.3 to 4.1)	.08
Hispanic	0.8 (−0.4 to 2.1)	.19	−0.4 (−1.6 to 0.8)	.48	0.7 (−1.1 to 2.5)	.43	0.4 (−1.4 to 2.2)	.67
Non-Hispanic Asian	0.7 (−1.8 to 3.2)	.56	1.2 (−1.0 to 3.4)	.27	2.5 (0.4 to 4.6)	.02	3.8 (1.2 to 6.3)	.005
Non-Hispanic other	0.3 (−1.8 to 2.3)	.79	−0.4 (−2.4 to 1.6)	.67	1.8 (−2.5 to 6.1)	.40	1.8 (−2.2 to 5.9)	.37
BMI^c^								
Normal weight	0 [Reference]	NA	0 [Reference]	NA	0 [Reference]	NA	0 [Reference]	NA
Overweight	3.1 (1.7 to 4.5)	<.001	3.2 (1.7 to 4.6)	<.001	2.6 (−0.1 to 5.1)	.04	3.2 (0.7 to 5.6)	.01
Obesity	6.8 (5.5 to 8.1)	<.001	6.8 (5.6 to 8.1)	<.001	3.4 (1.7 to 5.0)	<.001	3.5 (1.9 to 5.1)	<.001
PIR^d^								
≥3.50	0 [Reference]	NA	0 [Reference]	NA	0 [Reference]	NA	0 [Reference]	NA
1.30-3.49	2.1 (0.1 to 4.0)	.04	1.8 (−0.2 to 3.8)	.08	0.9 (−2.0 to 3.8)	.53	0.8 (−1.9 to 3.5)	.53
<1.30	2.3 (0.3 to 4.2)	.03	1.6 (−0.3 to 3.5)	.11	0.7 (−1.8 to 3.2)	.59	0.5 (−2.0 to 2.9)	.69
**Adolescents aged 13-17 y **								
Age, y								
13	0 [Reference]	NA	0 [Reference]	NA	0 [Reference]	NA	0 [Reference]	NA
14	1.8 (0.2 to 3.4)	.03	2.0 (0.5 to 3.6)	.01	0.0 (−2.5 to 2.5)	.98	0.1 (−2.8 to 2.9)	.97
15	3.1 (1.4 to 4.8)	.001	3.0 (1.2 to 4.8)	.002	3.6 (1.0 to 6.2)	.008	3.4 (0.7 to 6.1)	.02
16	3.7 (1.6 to 5.8)	.001	4.1 (1.9 to 6.2)	.001	4.0 (1.8 to 6.1)	.001	3.9 (1.7 to 6.2)	.001
17	5.0 (3.7 to 6.3)	<.001	5.2 (4.0 to 6.5)	<.001	5.2 (3.2 to 7.3)	<.001	5.6 (3.4 to 7.9)	<.001
Sex								
Female	0 [Reference]	NA	0 [Reference]	NA	0 [Reference]	NA	0 [Reference]	NA
Male	4.8 (3.6 to 6.0)	<.001	4.8 (3.8 to 5.8)	<.001	−1.7 (−3.2 to −0.1)	.03	−2.2 (−3.8 to 0.6)	.008
Race/ethnicity								
Non-Hispanic White	0 [Reference]	NA	0 [Reference]	NA	0 [Reference]	NA	0 [Reference]	NA
Non-Hispanic Black	4.0 (2.5 to 5.5)	<.001	3.0 (1.7 to 4.3)	<.001	−1.0 (−3.2 to 1.2)	.36	−0.8 (−3.1 to 1.5)	.48
Hispanic	1.3 (−0.3 to 2.9)	.10	0.4 (−1.1 to 1.9)	.58	−0.5 (−2.2 to 1.3)	.57	−0.4 (−2.3 to 1.5)	.68
Non-Hispanic Asian	−0.2 (−1.8 to 1.4)	.76	0.8 (−0.9 to 2.5)	.35	0.4 (−2.8 to 3.7)	.79	−0.03 (−3.6 to 3.6)	.99
Non-Hispanic other	−0.4 (−3.0 to 2.2)	.75	−0.4 (−2.6 to 1.9)	.74	−0.9 (−4.9 to 3.1)	.66	−1.0 (−5.4 to 3.4)	.65
BMI^c^								
Normal weight	0 [Reference]	NA	0 [Reference]	NA	0 [Reference]	NA	0 [Reference]	NA
Overweight	3.6 (2.1 to 5.1)	<.001	3.5 (1.9 to 5.1)	<.001	−1.4 (−3.2 to 0.5)	.14	−1.4 (−3.3 to 0.6)	.17
Obesity	6.6 (5.2 to 7.9)	<.001	6.6 (5.2 to 8.0)	<.001	0.5 (−1.8 to 2.9)	.64	0.7 (−1.8 to 3.2)	.60
PIR^d^								
≥3.50	0 [Reference]	NA	0 [Reference]	NA	0 [Reference]	NA	0 [Reference]	NA
1.30-3.49	1.2 (−0.3 to 2.6)	.11	1.0 (−0.6 to 2.6)	.20	0.8 (−1.3 to 3.0)	.45	0.8 (−1.1 to 2.8)	.40
<1.30	0.8 (−0.7 to 2.3)	.27	0.2 (−1.4 to 1.8)	.79	−0.1 (−2.5 to 2.2)	.90	0.2 (−2.1 to 2.4)	.88

Abbreviations: BMI, body mass index (calculated as weight in kilograms divided by height in meters squared); NA, not applicable; PIR, poverty-to-income ratio.

^a^ Adjusted for age, sex, and race/ethnicity.

^b^ Adjusted for variables in model 1, BMI, and PIR.

^c^ Normal indicates BMI of 5th percentile to less than 85th percentile; overweight, 85th percentile to less than 95th percentile; and obesity, 95th percentile or greater. Categories were based on age- and sex-specific growth charts developed by the Centers for Disease Control and Prevention in 2000. Owing to a small sample size, children with a BMI less than the 5th percentile are not presented.

^d^ A PIR <1.30 indicates that the family income was below 130% of the poverty level.

### Factors Associated With Hypertension and High BP in NHANES 2015-2018

Among children aged 8 to 12 years, those with obesity vs normal weight were more likely to have hypertension after multivariable adjustment (PR, 3.02; 95% CI, 1.78-5.11) (Table 3). Also, those with overweight (PR, 1.95; 95% CI, 1.33-2.87) or obesity (PR, 2.80; 95% CI, 2.04-3.84) vs normal weight or from a family with a middle (PR, 1.74; 95% CI, 1.02-2.97) or low (PR, 1.60; 95% CI, 1.01-2.55) PIR vs a high PIR were more likely to have high BP. Among adolescents aged 13 to 17 years, those who were aged 15 (PR for hypertension, 4.76 [95% CI, 1.47-15.46]; PR for high BP, 2.16 [95% CI, 1.24-3.76]), 16 (PR for hypertension, 5.62 [95% CI, 1.53-20.59]; PR for high BP, 2.78 [95% CI, 1.61-4.76]), or 17 (PR for hypertension, 4.97 [95% CI, 1.43-17.24]; PR for high BP, 3.37 [95% CI, 1.92-5.90]) years were more likely to have hypertension or high BP compared with those who were aged 13 years. Hypertension and high BP were more common among boys vs girls (PR for hypertension, 4.73 [95% CI, 2.10-10.68]; PR for high BP, 2.41 [95% CI, 1.59-3.64]), those who were non-Hispanic Black vs non-Hispanic White (PR for hypertension, 2.03 [95% IC, 1.01-4.07]; PR for high BP, 1.69 [95% CI, 1.16-2.46]), and those with overweight (PR for hypertension, 6.59 [95% CI, 2.76-15.72]; PR for high BP, 2.40 [95% CI, 1.47-3.92]) or obesity (PR for hypertension, 12.31 [95% CI, 4.62-32.74]; PR for high BP, 3.70 [95% CI, 2.67-5.13]) vs normal weight.

**Table 3.** Factors Associated With Hypertension and High Blood Pressure Among US Children and Adolescents in 2015-2018

Study participant characteristic	PR (95% CI)			
	Hypertension		High blood pressure	
	Model 1^a^	Model 2^b^	Model 1^a^	Model 2^b^
**Children aged 8-12 y **				
Age, y				
8	1 [Reference]	1 [Reference]	1 [Reference]	1 [Reference]
9	1.08 (0.49-2.35)	1.09 (0.51-2.31)	1.19 (0.69-2.04)	1.28 (0.78-2.11)
10	0.50 (0.19-1.34)	0.50 (0.19-1.30)	0.78 (0.43-1.42)	0.80 (0.47-1.38)
11	0.93 (0.49-1.75)	0.94 (0.51-1.72)	0.85 (0.53-1.35)	0.86 (0.56-1.32)
12	0.47 (0.19-1.16)	0.49 (0.19-1.29)	0.63 (0.39-1.36)	0.67 (0.30-1.50)
Sex				
Female	1 [Reference]	1 [Reference]	1 [Reference]	1 [Reference]
Male	0.96 (0.54-1.68)	0.95 (0.53-1.70)	1.00 (0.71-1.42)	1.01 (0.72-1.42)
Race/ethnicity				
Non-Hispanic White	1 [Reference]	1 [Reference]	1 [Reference]	1 [Reference]
Non-Hispanic Black	0.92 (0.43-1.98)	0.72 (0.30-1.77)	1.02 (0.72-1.45)	0.76 (0.49-1.20)
Hispanic	1.06 (0.63-1.81)	0.93 (0.50-1.71)	1.15 (0.77-1.71)	0.98 (0.70-1.36)
Non-Hispanic Asian	1.09 (0.46-2.59)	1.47 (0.63-3.43)	0.94 (0.46-1.93)	1.26 (0.67-2.36)
Non-Hispanic other	1.09 (0.49-2.41)	0.94 (0.37-2.43)	0.79 (0.38-1.64)	0.70 (0.31-1.59)
BMI^c^				
Normal weight	1 [Reference]	1 [Reference]	1 [Reference]	1 [Reference]
Overweight	1.54 (0.64-3.69)	1.51 (0.61-3.74)	1.99 (1.37-2.88)	1.95 (1.33-2.87)
Obesity	3.05 (1.78-5.20)	3.02 (1.78-5.11)	2.89 (2.09-4.01)	2.80 (2.04-3.84)
PIR^d^				
≥3.50	1 [Reference]	1 [Reference]	1 [Reference]	1 [Reference]
1.30-3.49	1.89 (0.69-5.20)	1.79 (0.66-4.81)	1.85 (1.05-3.27)	1.74 (1.02-2.97)
<1.30	1.78 (0.66-4.80)	1.55 (0.61-3.94)	1.83 (1.10-3.04)	1.60 (1.01-2.55)
**Adolescents aged 13-17 y**				
Age, y				
13	1 [Reference]	1 [Reference]	1 [Reference]	1 [Reference]
14	1.75 (0.47-6.54)	2.00 (0.52-7.75)	1.56 (0.82-2.95)	1.67 (0.88-3.17)
15	5.57 (1.83-16.94)	4.76 (1.47-15.46)	2.29 (1.38-3.79)	2.16 (1.24-3.76)
16	4.42 (1.32-14.79)	5.62 (1.53-20.59)	2.44 (1.38-4.32)	2.78 (1.61-4.76)
17	4.54 (1.49-13.83)	4.97 (1.43-17.24)	3.20 (1.89-5.42)	3.37 (1.92-5.90)
Sex				
Female	1 [Reference]	1 [Reference]	1 [Reference]	1 [Reference]
Male	4.87 (2.43-9.75)	4.73 (2.10-10.68)	2.49 (1.62-3.84)	2.41 (1.59-3.64)
Race/ethnicity				
Non-Hispanic White	1 [Reference]	1 [Reference]	1 [Reference]	1 [Reference]
Non-Hispanic Black	2.90 (1.32-6.36)	2.03 (1.01-4.07)	2.03 (1.42-2.89)	1.69 (1.16-2.46)
Hispanic	2.65 (1.18-5.94)	1.52 (0.65-3.51)	1.41 (0.92-2.17)	1.12 (0.75-1.67)
Non-Hispanic Asian	1.26 (0.36-4.43)	1.76 (0.39-7.94)	0.85 (0.46-1.56)	0.91 (0.48-1.71)
Non-Hispanic other	1.14 (0.34-3.76)	0.77 (0.21-2.77)	1.37 (0.74-2.53)	1.13 (0.71-1.81)
BMI^c^				
Normal weight	1 [Reference]	1 [Reference]	1 [Reference]	1 [Reference]
Overweight	5.63 (2.47-12.83)	6.59 (2.76-15.72)	2.30 (1.42-3.70)	2.40 (1.47-3.92)
Obesity	10.13 (4.38-23.41)	12.31 (4.62-32.74)	3.63 (2.65-4.97)	3.70 (2.67-5.13)
PIR^d^				
≥3.50	1 [Reference]	1 [Reference]	1 [Reference]	1 [Reference]
1.30–3.49	3.56 (1.13-11.19)	2.97 (0.95-9.29)	1.29 (0.83-1.98)	1.24 (0.77-2.01)
<1.30	1.79 (0.50-6.45)	1.36 (0.38-4.82)	1.21 (0.87-1.68)	1.08 (0.72-1.60)

Abbreviations: BMI, body mass index (calculated as weight in kilograms divided by height in meters squared); PIR, poverty-to-income ratio; PR, prevalence ratio.

^a^ Adjusted for age, sex, and race/ethnicity.

^b^ Adjusted for variables in model 1, BMI, and PIR.

^c^ Normal indicates BMI of 5th percentile to less than 85th percentile; overweight, 85th percentile to less than 95th percentile; and obesity, 95th percentile or greater. Categories were based on age- and sex-specific growth charts developed by the Centers for Disease Control and Prevention in 2000. Owing to a small sample size, children with a BMI less than the 5th percentile are not presented.

^d^ A PIR <1.30 indicates that the family income was below 130% of the poverty level.

## Discussion

In this analysis of data weighted to provide nationally representative estimates for US children and adolescents, mean SBP was lower in 2015-2018 than in 1999-2002 among adolescents aged 13 to 17 years, and mean DBP was lower in 2015-2018 than in 1999-2002 among children aged 8 to 12 years and adolescents aged 13 to 17 years. The prevalence of elevated BP was lower in 2015-2018 than in 1999-2002 among children aged 8 to 12 years, and the prevalence of hypertension was lower in 2015-2018 than in 1999-2002 among adolescents aged 13 to 17 years. However, mean SBP, mean DBP, and the proportion with hypertension did not decline from 2011-2014 to 2015-2018. Among children aged 8 to 12 years, differences in mean SBP and the prevalence of high BP and hypertension were small between sex and race/ethnicity groups in 2015-2018. However, among US adolescents aged 13 to 17 years, mean SBP and the prevalence of hypertension were higher among boys compared with girls and non-Hispanic Black compared with non-Hispanic White children and adolescents.

Similar to the association between BP and CVD in adults, it has been hypothesized that a linear relationship exists between SBP in childhood and subclinical CVD in adulthood.^15,16,17^ In a prior study,^18^ SBP at or above the age- and sex-specific 75th percentile for children and adolescents aged 12 to 18 years was associated with coronary artery calcification in adulthood. Levels of SBP in childhood and adolescence have also been associated with left ventricular hypertrophy and worse endothelial function in adulthood.^17,19^ This growing evidence base supports the benefit of maintaining optimal BP levels in early life and lowering the distribution of BP during childhood.^20,21,22^ Although a small reduction in mean SBP has occurred since 1999-2002 among adolescents aged 13 to 17 years, there is no evidence that mean SBP differed in 2015-2018 compared with 1999-2002 among children aged 8 to 12 years. Increased physical activity and improved dietary intake have been identified as primordial prevention strategies for lowering BP among children and adolescents.^23,24^ A recently published randomized clinical trial^25^ found that a 6-month Dietary Approaches to Stop Hypertension diet intervention lowered SBP by 2.7 mm Hg compared with routine care. Given BP tracking from childhood to adulthood,^4,26^ modest improvements in BP achievable through primordial prevention efforts in childhood could lower the risk of hypertension and CVD in adulthood.

In earlier work using NHANES data,^10^ the estimated prevalence of elevated BP decreased from 9.5% in 2005-2008 to 7.1% in 2013-2016, and the estimated prevalence of hypertension decreased from 5.7% in 2005-2008 to 3.5% in 2013-2016. Decreases in elevated BP and hypertension from 2003-2006 through 2011-2014 were present in the current study. However, similar to trends estimated in US adults,^11^ the estimated prevalence of elevated BP and hypertension and mean SBP and DBP have remained stable or potentially increased from 2011-2014 to 2015-2018. Future research should investigate whether changes in use of antihypertensives or other social, cardiometabolic, or environmental factors that influence BP have contributed to the trends in elevated BP and hypertension. Identifying and promoting improvements in modifiable factors that may have contributed to prior declines in hypertension trends could prevent or attenuate worsening in BP and hypertension among children and adolescents.

Consistent with prior studies that have documented differences in high BP and hypertension by race/ethnicity, a higher mean SBP and a higher prevalence of high BP and hypertension was estimated among non-Hispanic Black compared with non-Hispanic White adolescents aged 13 to 17 years.^27,28,29^ The emergence of disparities in BP between Black and White children during adolescence^29^ indicates that interventions in childhood could prevent the development of these disparities. However, little is known about the causes of racial/ethnic disparities in BP and hypertension among children and adolescents. Contributors to the higher prevalence of elevated BP and hypertension among Black compared with White adolescents are likely multifactorial and include sociodemographic,^30,31^ lifestyle,^29,32^ and physiological factors.^33^ In the present study, adjustment for BMI and PIR attenuated but did not fully account for the difference in hypertension and high BP between Black and White children and adolescents. Future studies should investigate the origins of racial/ethnic disparities in hypertension and implement strategies that increase health equity across populations.

A hypertension diagnosis in early childhood is generally considered secondary to an underlying disorder as opposed to essential or obesity-related hypertension.^34^ However, higher BP levels and a higher prevalence of hypertension were present in those with overweight or obesity compared with normal weight among both children aged 8 to 12 years and adolescents aged 13 to 17 years in this study. Prior studies have documented the effect of overweight or obesity on SBP and DBP in children as young as 2 to 5 years and indicate a strong association between BP and BMI in early life.^35,36,37^ Increases in BP and hypertension from 1988 to 2000 among both children and adolescents were partially attributed to increases in BMI.^38,39^ As the prevalence of obesity in the US continues to rise among children and adolescents,^40,41^ obesity-related increases in BP could further increase the prevalence of primary or obesity-related hypertension. Awareness of the effect of obesity on BP among children and effective interventions are needed to reduce the preventable development of hypertension among those aged 8 to 12 years.

### Limitations

This study has several limitations. First, 3 BP measurements were taken at a single visit, and guidelines recommend calculating a mean of multiple BP measurements obtained during 2 or more visits for diagnosing hypertension among children and adolescents.^12^ Second, the definition of hypertension relied solely on BP measurements or BP percentiles because data on treatment with antihypertensives were only available for NHANES participants aged 16 years or older. Third, the response rate for NHANES has declined from 1999-2002 through 2015-2018. However, any potential bias from the differential response rate across subgroups was reduced by weighting adjustment.^42^

## Conclusions

In this cross-sectional study from 1999-2002 to 2015-2018, mean SBP decreased among adolescents aged 13 to 17 years and mean DBP decreased among children aged 8 to 12 years and adolescents aged 13 to 17 years. The prevalence of elevated BP among children aged 8 to 12 years and the prevalence of hypertension among adolescents aged 13 to 17 years also decreased during this period. However, stable or increased BP levels and hypertension prevalence from 2011-2014 to 2015-2018 could indicate a reversal of these trends.

## Notes

Supplement. **eTable 1.** Definition of Normal Blood Pressure, Elevated Blood Pressure, and Hypertension **eTable 2.** Age-Adjusted Mean Systolic and Diastolic Blood Pressure Among US Children and Adolescents, Aged 8-12 and 13-17 Years, From 1999-2002 to 2015-2018 **eTable 3.** Systolic and Diastolic Blood Pressure Percentiles Among US Children and Adolescents, Aged 8-12 and 13-17 Years, From 1999-2002 to 2015-2018 **eTable 4.** Age-Adjusted Prevalence of Normal Blood Pressure, Elevated Blood Pressure and Hypertension Among US Children and Adolescents, Aged 8-12 and 13-17 Years, From 1999-2002 to 2015-2018 **eFigure.** Flowchart Showing the Number of NHANES Participants Included in the Current Analyses
