# Supplementary material for: Trends in Blood Pressure and Hypertension Among US Children and Adolescents, 1999-2018
Source: JAMA Netw Open. 2021 Apr 1;4(4):e213917. doi: 10.1001/jamanetworkopen.2021.3917 (PMC8017470; doi:10.1001/jamanetworkopen.2021.3917)
Supplement: Supplement. — eTable 1. Definition of Normal Blood Pressure, Elevated Blood Pressure, and Hypertension eTable 2. Age-Adjusted Mean Systolic and Diastolic Blood Pressure Among US Children and Adolescents, Aged 8-12 and 13-17 Years, From 1999-2002 to 2015-2018 eTable 3. Systolic and Diastolic Blood Pressure Percentiles Among US Children and Adolescents, Aged 8-12 and 13-17 Years, From 1999-2002 to 2015-2018 eTable 4. Age-Adjusted Prevalence of Normal Blood Pressure, Elevated Blood Pressure and Hypertension Among US Children and Adolescents, Aged 8-12 and 13-17 Years, From 1999-2002 to 2015-2018 eFigure. Flowchart Showing the Number of NHANES Participants Included in the Current Analyses [file jamanetwopen-e213917-s001.pdf]

Supplementary Online Content

Hardy ST, Sakhuja S, Jaeger BC, et al. Trends in blood pressure and hypertension among US children and adolescents, 1999-2018. *JAMA Netw Open*. 2021;4(4):e213917. doi:10.1001/jamanetworkopen.2021.3917

eTable 1. Definition of Normal Blood Pressure, Elevated Blood Pressure, and Hypertension

eTable 2. Age-Adjusted Mean Systolic and Diastolic Blood Pressure Among US Children and Adolescents, Aged 8-12 and 13-17 Years, From 1999-2002 to 2015-2018

eTable 3. Systolic and Diastolic Blood Pressure Percentiles Among US Children and Adolescents, Aged 8-12 and 13-17 Years, From 1999-2002 to 2015-2018

eTable 4. Age-Adjusted Prevalence of Normal Blood Pressure, Elevated Blood Pressure and Hypertension Among US Children and Adolescents, Aged 8-12 and 13-17 Years, From 1999-2002 to 2015-2018

eFigure. Flowchart Showing the Number of NHANES Participants Included in the Current Analyses

This supplementary material has been provided by the authors to give readers additional information about their work.

© 2021 Hardy ST et al. *JAMA Network Open*.

eTable 1. Definition of Normal Blood Pressure, Elevated Blood Pressure, and Hypertension

**Blood pressure category[*]**	**Children age 8 to 12 years**	**Children and adolescents age 13 to 17 years**
Normal blood pressure	SBP and DBP < 90th percentile[†]	SBP < 120 mm Hg and DBP < 80 mm Hg
Elevated blood pressure[§]	SBP and/or DBP ≥ 90th to < 95th percentile or<br><br>SBP ≥ 120 mm Hg and DBP < 80 mm Hg to the 95th percentile (whichever is lower)	SBP 120 to 129 mm Hg and DBP < 80 mm Hg
Hypertension[§]	SBP and/or DBP ≥ 95th percentile or<br><br>SBP ≥ 130 mm Hg and/or DBP ≥80 mm Hg	SBP ≥ 130 mm Hg and/or DBP ≥ 80 mm Hg

BP: blood pressure, SBP: systolic blood pressure, DBP: diastolic blood pressure
[*]Definitions were obtained from the 2017 American Academy of Pediatrics Clinical Practice Guideline
[†]Percentiles were determined using age-, sex-, and height-specific percentile tables from the 2017 American Academy of Pediatrics Clinical Practice Guideline.
[§]High blood pressure was defined as having either elevated blood pressure or hypertension.

© 2021 Hardy ST et al. *JAMA Network Open*.

eTable 2. Age-Adjusted Mean Systolic and Diastolic Blood Pressure Among US Children and Adolescents, Aged 8-12 and 13-17 Years, From 1999-2002 to 2015-2018

	NHANES Years						
	1999-2002	2003-2006	2007-2010	2011-2014	2015-2018	P trend*	P interaction
Age 8-12 Years							
Age-Adjusted Mean Systolic Blood Pressure, mm Hg (95% confidence interval)							
Overall	102.4 (101.7-103.1)	103.3 (102.4-104.1)	102.3 (101.4-103.2)	101.5 (100.8-102.2)	102.5 (101.9-103.2)	0.21	
Sex							0.88
Female	101.7 (100.9-102.5)	102.9 (101.7-104.1)	102.1 (100.9-103.3)	100.9 (100.1-101.6)	102.1 (101.2-102.9)	0.37	
Male	102.9 (101.9-103.9)	103.5 (102.6-104.5)	102.5 (101.6-103.4)	102.1 (101.3-102.9)	102.9 (101.3-103.5)	0.26	
Race							0.89
Non-Hispanic White	101.9 (101.0-102.8)	103.1 (101.9-104.3)	102.6 (101.5-103.7)	101.2 (100.2-102.1)	102.1 (101.1-103.0)	0.37	
Non-Hispanic Black	103.4 (102.6-104.2)	103.7 (102.5-105.0)	103.1 (102.0-104.2)	102.6 (101.5-103.6)	103.0 (101.9-104.1)	0.28	
Hispanic	102.7 (101.5-103.9)	103.7 (102.7-104.8)	101.5 (100.1-103.0)	101.5 (100.6-102.4)	102.9 (101.9-103.9)	0.53	
Non-Hispanic Asian†	-	-	-	100.9 (99.3-102.4)	103.0 (100.8-105.2)	0.14	
Non-Hispanic other	104.2 (102.1-106.3)	101.7 (99.1-104.4)	101.4 (99.4-103.6)	102.6 (100.9-104.4)	102.1 (100.4-103.8)	0.48	
Body mass index‡							0.87
Normal	100.7 (100.0-101.4)	101.5 (100.6-102.5)	100.6 (99.7-101.4)	99.9 (99.1-100.7)	100.7 (100.0-101.5)	0.26	
Overweight	105.6 (104.5-106.6)	104.0 (102.4-105.6)	104.1 (102.6-105.7)	102.4 (101.3-103.5)	103.8 (102.4-105.2)	0.01	
Obese	106.9 (105.2-108.5)	108.8 (107.6-110.1)	106.9 (105.8-108.1)	107.5 (105.4-108.2)	107.5 (106.7-108.4)	0.75	
Poverty-to-income ratio§							0.40
<1.30	102.8 (101.6-103.9)	103.6 (102.6-104.7)	102.5 (101.6-103.4)	101.9 (100.9-102.9)	103.3 (102.4-104.2)	0.78	
1.30–3.49	102.2 (101.4-103.1)	103.9 (102.7-105.0)	102.7 (101.7-103.7)	102.0 (101.1-102.9)	103.0 (102.2-103.9)	0.75	
≥3.50	101.6 (100.4-102.8)	102.1 (100.8-103.4)	102.2 (100.9-103.4)	100.7 (99.4-102.1)	100.9 (99.4-102.5)	0.22	
Age-Adjusted Mean Diastolic Blood Pressure, mm Hg (95% confidence interval)							
Overall	57.2 (56.5-58.0)	54.3 (53.2-55.5)	53.0 (51.6-54.4)	51.9 (50.1-53.7)	53.2 (52.2-54.1)	<0.001	
Sex							0.06
Female	57.4 (56.5-58.2)	54.5 (53.2-55.9)	54.3 (52.8-55.8)	53.1 (51.2-55.0)	54.0 (52.6-55.4)	<0.001	
Male	57.1 (56.1-58.1)	54.2 (52.9-55.5)	51.7 (50.1-53.2)	50.7 (48.7-52.8)	52.4 (51.2-53.7)	<0.001	
Race							0.96
Non-Hispanic White	57.0 (55.9-58.1)	53.9 (52.3-55.6)	53.1 (51.2-54.9)	52.8 (50.3-55.4)	52.5 (51.2-53.8)	<0.001	
Non-Hispanic Black	57.4 (55.8-59.0)	54.9 (53.6-56.3)	54.1 (51.8-56.3)	50.4 (48.3-52.5)	54.5 (52.4-56.6)	0.001	
Hispanic	57.5 (56.3-58.7)	55.1 (53.9-56.3)	52.4 (51.0-53.7)	50.3 (48.2-52.5)	53.2 (51.9-54.4)	<0.001	
Non-Hispanic Asian	-	-	-	52.2 (50.2-54.2)	55.0 (53.1-57.0)	0.06	
Non-Hispanic other	59.6 (55.9-63.3)	53.9 (51.6-56.3)	52.3 (48.6-56.3)	52.3 (48.7-55.9)	54.3 (50.8-57.7)	0.10	
Body mass index							0.15
Normal	56.6 (55.6-57.5)	54.1 (52.9-55.4)	51.4 (49.8-53.1)	51.2 (49.5-52.9)	52.0 (50.7-53.3)	<0.001	

© 2021 Hardy ST et al. *JAMA Network Open*.

Overweight	58.3 (56.5-60.1)	53.2 (51.5-54.9)	54.3 (52.6-56.0)	51.5 (49.2-53.7)	54.6 (52.7-56.5)	0.006	
Obese	58.9 (57.3-60.5)	56.1 (53.9-58.3)	57.0 (55.3-58.7)	55.2 (52.4-57.9)	55.4 (53.9-56.9)	0.009	
Poverty-to-income ratio							0.87
<1.30	57.5 (56.4-58.6)	53.8 (52.0-55.7)	53.6 (52.1-55.0)	51.2 (48.8-53.5)	53.6 (52.2-54.9)	<0.001	
1.30–3.49	57.0 (55.7-58.3)	54.5 (53.4-55.6)	52.9 (50.5-55.3)	52.3 (50.5-54.0)	53.5 (52.0-55.0)	<0.001	
≥3.50	57.2 (55.8-58.6)	54.3 (52.2-56.4)	52.6 (51.0-54.3)	52.8 (50.1-55.6)	52.5 (50.4-54.5)	0.001	
Age 13-17 Years							
Age-Adjusted Mean Systolic Blood Pressure, mm Hg (95% confidence interval)							
Overall	109.2 (108.7-109.7)	109.7 (108.9-110.4)	108.5 (107.7-109.3)	108.4 (107.8-109.1)	108.4 (107.8-109.1)	0.007	
Sex							0.92
Female	106.7 (105.9-107.5)	107.4 (106.5-108.3)	105.9 (105.0-106.7)	105.9 (105.1-106.6)	106.0 (105.3-106.8)	0.03	
Male	111.7 (110.9-112.5)	112.0 (111.1-112.9)	111.1 (110.1-112.1)	111.1 (110.1-112.0)	110.9 (109.8-111.9)	0.06	
Race							0.59
Non-Hispanic White	108.5 (107.8-109.3)	109.8 (108.7-110.8)	108.3 (107.2-109.4)	107.9 (107.0-108.8)	107.6 (106.7-108.6)	0.007	
Non-Hispanic Black	111.7 (110.7-112.7)	111.1 (110.4-111.8)	110.6 (109.5-111.7)	110.2 (109.5-110.9)	111.8 (110.6-113.0)	0.71	
Hispanic	109.4 (108.6-110.3)	109.3 (108.3-110.2)	108.1 (107.0-109.1)	108.8 (107.5-110.0)	108.9 (107.7-110.1)	0.42	
Non-Hispanic Asian	-	-	-	107.3 (105.9-108.6)	107.4 (105.5-109.2)	0.94	
Non-Hispanic other	109.6 (107.0-112.1)	106.2 (104.8-107.6)	107.1 (104.2-110.0)	107.4 (105.1-109.7)	107.4 (105.0-109.5)	0.43	
Body mass index							0.08
Normal	107.4 (106.8-107.9)	108.2 (107.2-109.2)	107.0 (106.1-107.9)	106.8 (105.8-107.8)	106.5 (105.8-107.2)	0.007	
Overweight	111.4 (109.8-112.9)	110.7 (109.6-111.7)	110.8 (109.1-112.5)	110.7 (109.4-112.1)	109.7 (108.5-111.0)	0.12	
Obese	116.0 (114.9-117.1)	115.3 (114.3-116.4)	112.6 (111.3-113.9)	111.7 (110.5-112.9)	113.7 (112.3-115.1)	<0.001	
Poverty-to-income ratio							0.41
<1.30	110.0 (108.9-111.1)	109.3 (108.4-110.3)	108.7 (107.6-109.8)	109.0 (108.2-109.7)	108.8 (107.7-110.0)	0.10	
1.30–3.49	108.9 (108.2-109.6)	110.1 (109.0-111.2)	108.7 (107.7-109.8)	108.7 (107.8-109.6)	109.0 (107.8-110.0)	0.34	
≥3.50	108.6 (107.7-109.5)	109.4 (108.2-110.5)	108.0 (106.5-109.5)	107.6 (106.2-109.1)	107.1 (106.1-108.2)	0.008	
Age-Adjusted Mean Diastolic Blood Pressure, mm Hg (95% confidence interval)							
Overall	62.6 (61.7-63.5)	59.7 (58.9-60.4)	59.8 (58.4-61.2)	59.6 (58.2-60.9)	60.8 (59.8-61.7)	0.03	
Sex							0.02
Female	64.2 (63.0-65.4)	61.6 (60.9-62.3)	61.1 (59.7-62.4)	60.7 (59.1-62.4)	61.6 (60.6-62.7)	0.003	
Male	60.9 (59.9-61.9)	57.8 (56.8-58.8)	58.6 (56.8-60.3)	58.4 (57.0-59.9)	59.9 (58.7-61.2)	0.66	
Race							0.39
Non-Hispanic White	62.8 (61.8-63.9)	59.9 (58.9-60.8)	60.3 (58.7-61.8)	60.6 (58.7-62.6)	61.1 (59.7-62.4)	0.22	
Non-Hispanic Black	61.4 (60.2-62.7)	59.8 (58.8-60.7)	60.7 (58.7-62.7)	57.8 (55.6-59.9)	59.9 (58.0-61.9)	0.08	
Hispanic	61.7 (60.2-63.1)	59.0 (57.8-60.1)	57.5 (55.5-59.5)	58.5 (57.1-60.0)	60.6 (59.4-61.8)	0.31	
Non-Hispanic Asian	-	-	-	58.9 (56.5-61.2)	61.5 (58.6-64.4)	0.17	
Non-Hispanic other	65.3 (61.8-68.7)	59.3 (56.9-61.7)	60.2 (57.0-63.3)	60.6 (58.3-62.8)	60.6 (57.6-63.6	0.09	

© 2021 Hardy ST et al. *JAMA Network Open*.

Body mass index							0.07
Normal	62.8 (61.7-64.0)	60.0 (59.2-60.9)	59.8 (58.4-61.3)	59.4 (58.0-60.8)	60.9 (59.8-62.0)	0.02	
Overweight	61.4 (60.2-62.7)	57.8 (56.2-59.4)	58.6 (56.2-60.9)	58.3 (56.2-60.5)	59.6 (57.9-61.3)	0.38	
Obese	61.5 (59.9-63.1)	59.8 (58.3-61.3)	60.6 (58.5-62.8)	61.0 (58.5-63.1)	61.1 (59.2-62.9)	0.78	
Poverty-to-income ratio							0.92
<1.30	61.9 (60.2-63.4)	59.4 (58.4-60.4)	59.4 (57.7-61.1)	58.5 (56.7-60.2)	60.3 (58.9-61.6)	0.08	
1.30–3.49	62.9 (61.7-64.1)	58.8 (57.7-59.9)	59.6 (58.1-61.1)	59.7 (57.5-61.9)	61.2 (59.9-62.6)	0.59	
≥3.50	62.9 (62.0-63.7)	60.7 (59.5-61.8)	60.3 (58.1-62.5)	60.3 (58.6-62.1)	60.7 (59.2-62.1)	0.04	

NHANES: National Health and Nutrition Examination Survey

[*]P value for trend in age-adjusted blood pressure levels across survey years.

[†] Non-Hispanic Asians were not presented in 1999-2002, 2003-2006, and 2007-2010 due to small sample sizes.

[‡] Body mass index was categorized as normal (body mass index 5th percentile to < 85th percentile), overweight (body mass index 85th percentile to < 95th percentile) and obese (body mass index ≥ 95th percentile) based on age- and sex-specific growth charts developed by the Centers for Disease Control and Prevention in 2000. Due to a small sample size, children with a body mass index < 5th percentile are not presented.

[§]A poverty-to-income ratio < 1.30 indicates that the family income was below 130% of the poverty level.

Age-adjustment was performed using direct standardization with the standard being US children and adolescents across the entire time period from 1999 to 2018.

© 2021 Hardy ST et al. *JAMA Network Open*.

eTable 3. Systolic and Diastolic Blood Pressure Percentiles Among US Children and Adolescents, Aged 8-12 and 13-17 Years, From 1999-2002 to 2015-2018

	NHANES Years					
	1999-2002	2003-2006	2007-2010	2011-2014	2015-2018	P trend*
Age 8-12 Years						
Systolic Blood Pressure, mm Hg						
5th percentile	88.0 (86.7, 89.3)	88.7 (87.6, 89.7)	88.0 (86.4, 89.6)	88.7 (87.7, 89.6)	88.7 (87.4, 89.9)	0.31
15th percentile	93.3 (92.1, 94.6)	93.3 (92.4, 94.3)	93.0 (91.7, 94.3)	92.7 (92.0, 93.3)	94.0 (93.4, 94.6)	0.73
25th percentile	96.7 (95.7, 97.6)	96.7 (95.4, 97.9)	96.0 (95.0, 97.0)	95.3 (94.4, 96.3)	96.7 (96.0, 97.3)	0.56
50th percentile	102.0 (101.4, 102.6)	102.0 (101.0, 103.0)	102.0 (101.0, 103.0)	100.7 (100.0, 101.3)	102.0 (101.4, 102.6)	0.56
75th percentile	107.3 (106.4, 108.3)	110.0 (108.7, 111.3)	108.0 (107.0, 109.0)	107.3 (106.2, 108.4)	108.0 (107.4, 108.6)	0.76
85th percentile	111.3 (110.0, 112.6)	113.3 (112.1, 114.6)	112.0 (111.0, 113.0)	111.3 (110.4, 112.3)	111.3 (110.4, 112.3)	0.55
95th percentile	117.8 (116.2, 119.3)	119.3 (117.8, 120.7)	118.7 (116.4, 120.9)	117.3 (116.4, 118.3)	117.3 (116.1, 118.6)	0.37
Diastolic Blood Pressure, mm Hg						
5th percentile	38.0 (34.8, 41.2)	34.0 (31.4, 36.6)	28.5 (23.4, 33.7)	23.3 (19.2, 27.5)	28.7 (25.5, 31.9)	0.09
15th percentile	46.7 (45.7, 47.6)	42.7 (41.4, 43.9)	40.7 (39.1, 42.3)	38.7 (34.8, 42.5)	42.0 (40.1, 43.9)	0.18
25th percentile	50.7 (49.7, 51.6)	47.3 (45.6, 49.0)	46.0 (44.4, 47.6)	44.7 (42.7, 46.6)	46.7 (45.4, 47.9)	0.14
50th percentile	58.0 (57.0, 59.0)	55.3 (54.4, 56.3)	54.0 (52.4, 55.6)	54.0 (52.1, 55.9)	54.7 (53.7, 55.6)	0.13
75th percentile	65.3 (64.7, 66.0)	62.0 (61.0, 63.0)	62.0 (60.6, 63.4)	61.3 (59.4, 63.3)	62.0 (60.8, 63.2)	0.16
85th percentile	68.0 (67.0, 69.0)	66.0 (65.0, 67.0)	66.0 (64.9, 67.1)	65.3 (63.4, 67.3)	66.0 (65.0, 67.0)	0.16
95th percentile	73.3 (72.7, 74.0)	71.3 (69.7, 73.0)	72.0 (70.7, 73.3)	71.3 (69.1, 73.6)	72.0 (70.4, 73.6)	0.37
Age 13-17 Years						
Systolic Blood Pressure, mm Hg						
5th percentile	92.8 (91.2, 94.4)	94.7 (93.7, 95.6)	92.0 (90.6, 93.4)	93.3 (91.7, 94.9)	94.0 (92.7, 95.3)	0.80
15th percentile	98.7 (97.7, 99.6)	100.0 (99.2, 100.8)	98.7 (97.4, 99.9)	98.7 (97.1, 100.3)	98.7 (97.4, 99.9)	0.56
25th percentile	102.0 (101.4, 102.6)	103.0 (102.0, 104.0)	101.3 (100.1, 102.6)	102.2 (101.2, 103.1)	102.0 (101.0, 103.0)	0.72
50th percentile	108.7 (108.0, 109.3)	109.3 (108.4, 110.3)	108.0 (107.0, 109.0)	108.0 (107.4, 108.6)	108.0 (107.0, 109.0)	0.18
75th percentile	116.0 (115.2, 116.8)	116.0 (115.1, 116.9)	114.7 (113.4, 115.9)	114.7 (114.3, 115.0)	114.7 (113.7, 115.6)	0.06
85th percentile	119.3 (118.7, 120.0)	119.3 (118.1, 120.6)	118.7 (118.0, 119.3)	118.0 (116.7, 119.3)	118.0 (117.4, 118.6)	0.01
95th percentile	126.7 (125.4, 127.9)	127.3 (125.1, 129.5)	126.0 (124.1, 127.9)	124.0 (122.4, 125.6)	124.7 (123.4, 125.9)	0.07
Diastolic Blood Pressure, mm Hg						
5th percentile	45.0 (42.4, 47.5)	41.3 (39.7, 42.9)	40.7 (35.2, 46.2)	39.3 (35.6, 43.0)	43.3 (41.2, 45.4)	0.53
15th percentile	52.0 (51.0, 53.0)	49.0 (48.0, 50.0)	50.0 (48.4, 51.6)	48.7 (46.7, 50.6)	50.0 (48.7, 51.3)	0.36
25th percentile	56.0 (54.7, 57.3)	53.3 (52.4, 54.3)	54.0 (52.1, 55.9)	53.3 (51.8, 54.9)	54.7 (53.4, 55.9)	0.53
50th percentile	63.3 (62.4, 64.3)	60.0 (59.4, 60.6)	60.7 (59.4, 61.9)	60.7 (59.1, 62.3)	62.0 (61.0, 63.0)	0.70
75th percentile	70.0 (69.4, 70.6)	67.3 (66.4, 68.3)	67.1 (65.9, 68.4)	67.3 (65.9, 68.7)	68.0 (67.0, 69.0)	0.35
85th percentile	73.3 (72.4, 74.3)	71.3 (70.4, 72.3)	70.0 (68.4, 71.6)	71.3 (70.4, 72.3)	71.3 (70.7, 72.0)	0.36

© 2021 Hardy ST et al. *JAMA Network Open*.

95th percentile	78.0 (76.7, 79.3)	76.0 (75.4, 76.6)	76.7 (75.4, 77.9)	76.0 (75.0, 77.0)	75.3 (74.4, 76.3)	0.08

Age-adjustment was performed using direct standardization with the standard being US children and adolescents across the entire time period from 1999 to 2018.

© 2021 Hardy ST et al. *JAMA Network Open*.

eTable 4. Age-Adjusted Prevalence of Normal Blood Pressure, Elevated Blood Pressure and Hypertension Among US Children and Adolescents, Aged 8-12 and 13-17 Years, From 1999-2002 to 2015-2018

	NHANES Years						
	1999-2002	2003-2006	2007-2010	2011-2014	2015-2018	P trend*	P interaction
Age 8-12 Years							
Age-Adjusted Normal Blood Pressure, Percent (95% Confidence Interval)							
Overall	87.8 (85.1-90.5)	86.5 (83.6-89.4)	88.5 (85.9-91.1)	91.0 (89.4-92.6)	89.9 (88.0-91.7)	0.02	
Sex							0.19
Female	90.0 (87.4-92.6)	87.8 (84.2-91.4)	87.9 (84.0-91.7)	92.1 (90.1-94.1)	89.8 (87.1-92.6)	0.35	
Male	86.0 (82.5-89.6)	85.4 (81.7-89.1)	89.1 (86.4-91.7)	90.0 (87.6-92.4)	89.8 (87.5-92.1)	0.01	
Race							0.50
Non-Hispanic White	88.8 (85.4-92.2)	86.5 (81.9-91.0)	88.4 (85.0-91.7)	91.0 (88.6-93.4)	90.2 (87.6-92.9)	0.17	
Non-Hispanic Black	86.5 (82.5-90.4)	86.5 (82.9-90.0)	88.7 (85.2-92.1)	90.9 (88.6-93.3)	90.1 (86.2-94.0)	0.07	
Hispanic	86.3 (81.4-91.3)	86.0 (82.2-89.8)	88.8 (84.3-93.3)	91.2 (88.6-93.8)	88.6 (85.3-91.8)	0.17	
Non-Hispanic Asian†	.	.	.	94.3 (90.7-97.9)	90.5 (84.9-96.1)	0.28	
Non-Hispanic other	83.8 (73.6-94.0)	89.2 (80.1-98.3)	88.0 (80.4-95.7)	87.0 (80.6-93.4)	93.4 (89.0-97.9)	0.24	
Body mass index‡							0.61
Normal	91.3 (88.7-93.9)	89.1 (84.9-93.3)	91.4 (89.0-93.8)	94.4 (92.7-96.1)	93.3 (91.9-94.7)	0.01	
Overweight	83.6 (78.0-89.3)	90.4 (86.0-94.8)	87.3 (83.1-91.5)	89.7 (86.3-93.0)	86.8 (82.0-91.6)	0.46	
Obese	76.4 (70.0-82.7)	73.7 (67.9-79.6)	81.1 (77.8-86.3)	81.2 (76.0-86.4)	80.9 (77.1-84.7)	0.08	
Poverty-to-income ratio§							0.51
<1.30	87.8 (83.0-92.5)	85.8 (82.5-89.0)	86.8 (83.5-89.0)	89.9 (87.0-92.8)	87.5 (84.8-90.2)	0.63	
1.30–3.49	85.4 (81.8-89.0)	85.1 (80.2-90.1)	89.6 (85.9-93.4)	90.8 (87.2-94.4)	87.7 (85.0-90.3)	0.07	
≥3.50	92.1 (88.9-95.2)	89.0 (83.3-94.7)	88.3 (84.3-92.2)	93.0 (90.1-96.0)	93.4 (90.2-96.6)	0.28	
Age-Adjusted Elevated Blood Pressure, Percent (95% Confidence Interval)							
Overall	7.0 (5.4-8.6)	7.3 (5.6-9.0)	6.5 (4.7-8.3)	4.3 (3.0-5.6)	5.5 (3.9-7.0)	0.02	
Sex							0.73
Female	6.0 (4.2-7.8)	6.4 (3.6-9.1)	6.3 (3.7-8.9)	3.3 (1.9-4.7)	5.4 (3.3-7.6)	0.18	
Male	7.8 (5.6-9.9)	8.0 (5.6-10.5)	6.9 (5.0-8.7)	5.2 (3.4-7.0)	5.7 (4.0-7.4)	0.03	
Race							0.56
Non-Hispanic White	6.6 (4.3-8.9)	7.9 (5.2-10.5)	6.6 (4.2-9.0)	4.1 (1.9-6.2)	5.3 (2.5-8.0)	0.14	
Non-Hispanic Black	7.9 (5.5-10.3)	6.6 (3.9-9.2)	5.4 (3.2-7.6)	6.1 (4.1-8.0)	5.5 (3.3-7.8)	0.29	
Hispanic	6.9 (3.7-10.2)	7.6 (4.6-10.6)	6.4 (3.2-9.6)	4.8 (3.2-6.4)	6.5 (4.2-8.8)	0.44	
Non-Hispanic Asian	.	.	.	0.9 (-0.3-2.2)	4.4 (0.2-8.6)	0.06	
Non-Hispanic other	10.8 (2.8-18.9)	4.4 (-0.1-9.0)	8.6 (2.3-14.9)	2.1 (-0.1-4.3)	2.4 (-0.01-4.8)	0.08	
Body mass index							0.12

© 2021 Hardy ST et al. *JAMA Network Open*.

Normal	5.2 (3.6-6.7)	6.3 (4.0-8.7)	5.1 (3.2-6.9)	2.1 (1.2-3.0)	3.4 (2.1-4.8)	0.002	
Overweight	11.3 (6.3-16.3)	5.7 (2.4-8.9)	6.9 (3.6-10.2)	5.5 (2.6-8.4)	8.3 (4.7-11.8)	0.35	
Obese	10.8 (7.3-14.3)	12.5 (7.3-14.3)	10.8 (6.5-15.0)	9.5 (5.3-13.7)	9.4 (6.0-12.8)	0.41	
Poverty-to-income ratio							0.19
<1.30	6.3 (3.9-8.7)	7.5 (5.0-10.1)	5.2 (2.9-7.6)	5.2 (3.2-7.2)	7.2 (4.7-9.6)	0.83	
1.30–3.49	9.0 (6.1-11.8)	7.5 (4.6-10.3)	5.8 (4.0-7.7)	4.6 (2.5-6.8)	6.7 (4.7-8.7)	0.05	
≥3.50	5.0 (2.6-7.3)	7.0 (3.2-10.9)	8.7 (4.7-12.7)	2.3 (0.3-4.2)	3.5 (0.5-6.6)	0.14	
Age-Adjusted Hypertension, Percent (95% Confidence Interval)							
Overall	5.2 (3.4-6.9)	6.2 (4.3-8.1)	5.0 (3.6-6.3)	4.7 (3.6-5.9)	4.6 (3.4-5.9)	0.30	
Sex							0.20
Female	4.0 (2.1-5.9)	5.9 (3.6-8.1)	5.9 (3.7-8.0)	4.6 (2.9-6.3)	4.7 (2.8-6.6)	0.90	
Male	6.2 (3.8-8.6)	6.5 (3.7-9.3)	4.1 (2.6-5.5)	4.8 (2.7-6.9)	4.5 (3.0-6.0)	0.12	
Race							0.68
Non-Hispanic White	4.6 (2.4-6.8)	5.7 (3.0-8.3)	5.0 (2.9-7.2)	4.9 (3.2-6.7)	4.5 (2.8-6.3)	0.82	
Non-Hispanic Black	5.6 (3.3-7.9)	7.0 (5.1-8.9)	5.9 (3.4-8.4)	3.0 (1.7-4.3)	4.3 (1.5-7.3)	0.11	
Hispanic	6.7 (3.8-9.7)	6.4 (4.2-8.7)	4.8 (3.1-6.5)	4.0 (2.2-5.8)	4.9 (3.1-6.7)	0.15	
Non-Hispanic Asian	.	.	.	4.7 (1.6-7.8)	5.1 (1.8-8.3)	>0.99	
Non-Hispanic other	5.4 (-0.8-11.5)	6.3 (-0.3-13.0)	3.4 (-0.3-7.0)	10.9 (4.4-17.0)	4.2 (0.6-7.8)	0.94	
Body mass index							0.56
Normal	3.5 (1.7-5.3)	4.6 (2.0-7.2)	3.5 (1.8-5.1)	3.5 (1.9-5.1)	3.2 (2.0-4.5)	0.55	
Overweight	5.1 (1.9-8.2)	3.9 (1.7-6.1)	5.8 (2.1-9.5)	4.8 (3.2-6.4)	4.9 (1.1-8.7)	0.94	
Obese	12.8 (7.4-18.2)	13.8 (9.0-18.6)	8.2 (5.1-11.2)	9.3 (4.9-13.7)	9.7 (6.7-12.7)	0.13	
Poverty-to-income ratio							0.68
<1.30	5.9 (2.4-9.5)	6.7 (4.8-8.6)	7.9 (6.0-9.8)	4.9 (2.8-7.0)	5.4 (3.4-7.4)	0.63	
1.30–3.49	5.6 (3.6-7.7)	7.4 (3.3-11.4)	4.5 (2.1-7.0)	4.5 (1.9-7.2)	5.7 (3.6-7.7)	0.48	
≥3.50	2.9 (1.3-4.6)	4.0 (1.3-6.6)	3.1 (0.8-5.3)	4.7 (2.3-7.0)	3.0 (0.8-5.3)	0.83	
Age 13-17 Years							
Age-Adjusted Normal Blood Pressure, Percent (95% Confidence Interval)							
Overall	83.8 (82.0-85.6)	83.9 (81.3-86.4)	85.9 (83.4-88.3)	87.6 (85.2-90.0)	87.9 (85.9-89.9)	<0.001	
Sex							0.31
Female	90.2 (87.5-92.9)	89.8 (87.2-92.4)	92.8 (90.9-94.6)	94.1 (92.1-96.2)	93.1 (90.7-95.4)	0.01	
Male	77.5 (75.0-80.1)	78.1 (74.8-81.5)	79.1 (75.6-82.6)	80.5 (76.0-85.1)	82.7 (79.3-86.1)	0.02	
Race							0.19
Non-Hispanic White	84.9 (82.1-87.6)	83.8 (80.5-87.2)	86.6 (83.1-90.2)	90.0 (86.3-93.6)	90.4 (87.3-93.6)	0.001	
Non-Hispanic Black	78.5 (74.8-82.2)	79.6 (76.8-82.4)	79.9 (75.9-83.8)	82.0 (78.8-85.2)	79.8 (76.2-83.3)	0.36	
Hispanic	85.8 (82.6-89.0)	85.1 (81.8-88.4)	87.4 (84.6-90.2)	84.7 (80.6-88.9)	86.6 (83.4-89.7)	0.82	
Non-Hispanic Asian	.	.	.	90.3 (86.0-94.6)	91.6 (86.7-96.5)	0.59	

© 2021 Hardy ST et al. *JAMA Network Open*.

Non-Hispanic other	81.6 (70.8-92.5)	92.5 (89.3-95.7)	87.4 (77.7-97.2)	87.9 (79.3-96.5)	85.8 (79.9-91.7)	0.75	
Body mass index							0.46
Normal	88.1 (85.7-90.4)	87.6 (84.3-90.9)	90.6 (88.3-92.9)	90.9 (88.5-93.3)	93.3 (91.5-95.1)	<0.001	
Overweight	81.4 (75.9-86.9)	82.9 (79.2-86.9)	80.6 (74.2-87.0)	83.6 (77.6-89.7)	86.0 (80.8-91.2)	0.31	
Obese	65.0 (60.3-69.7)	69.1 (62.8-75.4)	74.0 (69.1-78.9)	79.7 (75.1-84.4)	73.5 (68.0-79.1)	0.007	
Poverty-to-income ratio							0.07
<1.30	83.5 (79.9-87.1)	86.6 (83.7-89.6)	86.6 (83.7-89.5)	86.4 (82.9-89.8)	86.2 (83.4-89.0)	0.31	
1.30–3.49	84.3 (81.5-87.1)	82.5 (79.5-85.6)	87.0 (83.7-90.2)	87.6 (84.7-90.6)	86.3 (82.6-90.0)	0.08	
≥3.50	84.1 (81.2-87.1)	83.7 (79.8-87.7)	85.2 (80.9-89.6)	87.6 (82.7-92.6)	90.9 (88.1-93.8)	0.003	
Age-Adjusted Elevated Blood Pressure, Percent (95% Confidence Interval)							
Overall	9.5 (8.0-11.0)	10.7 (8.9-12.4)	9.5 (7.5-11.4)	9.9 (7.8-11.9)	8.4 (6.7-10.1)	0.23	
Sex							0.81
Female	5.3 (3.7-6.9)	6.4 (4.6-8.2)	4.9 (3.2-6.6)	4.7 (2.7-6.8)	5.7 (3.4-8.0)	0.75	
Male	13.8 (11.4-16.1)	14.8 (12.1-17.5)	14.0 (10.8-17.2)	15.4 (11.6-19.3)	11.3 (8.7-13.9)	0.24	
Race							0.27
Non-Hispanic White	9.2 (7.0-11.3)	10.1 (7.9-12.2)	8.4 (6.1-10.8)	8.4 (5.2-11.6)	7.3 (4.7-9.9)	0.16	
Non-Hispanic Black	12.4 (9.5-15.3)	14.5 (11.9-17.1)	13.2 (9.4-16.9)	13.3 (10.5-16.0)	13.5 (8.9-18.0)	0.94	
Hispanic	9.2 (6.5-11.9)	11.5 (8.5-14.4)	9.5 (6.8-12.1)	12.4 (8.8-16.2)	7.7 (5.5-9.8)	0.56	
Non-Hispanic Asian	.	.	.	6.6 (3.1-10.2)	5.5 (2.0-8.9)	0.64	
Non-Hispanic other	7.1 (3.6-11.0)	4.9 (0.2-9.5)	11.8 (2.1-21.4)	7.6 (0.9-14.4)	11.0 (4.0-18.1)	0.18	
Body mass index							0.84
Normal	7.1 (5.8-8.3)	8.2 (6.0-10.4)	6.2 (4.2-8.3)	7.3 (5.0-9.5)	5.8 (4.0-7.5)	0.22	
Overweight	14.0 (9.6-18.3)	10.0 (6.8-13.2)	13.3 (7.4-19.1)	14.4 (8.7-20.2)	9.4 (4.9-13.9)	0.52	
Obese	17.8 (14.1-21.6)	21.5 (15.7-27.4)	17.0 (11.7-22.4)	15.0 (10.3-19.7)	15.7 (11.3-20.0)	0.14	
Poverty-to-income ratio							0.62
<1.30	11.0 (8.0-14.0)	9.9 (7.6-12.2)	9.7 (7.4-12.1)	10.5 (7.4-13.3)	10.5 (8.1-12.9)	0.93	
1.30–3.49	9.4 (7.0-11.8)	11.4 (8.7-14.1)	7.8 (5.2-10.4)	10.8 (7.7-13.8)	8.2 (5.5-11.0)	0.38	
≥3.50	8.6 (6.2-11.0)	10.5 (7.6-13.4)	10.6 (6.6-14.5)	9.2 (5.1-13.2)	7.9 (5.2-10.5)	0.54	
Age-Adjusted Hypertension, Percent (95% Confidence Interval)							
Overall	6.6 (5.6-7.7)	5.5 (4.2-6.8)	4.6 (3.2-6.0)	2.5 (1.6-3.5)	3.7 (2.6-4.7)	<0.001	
Sex							0.03
Female	4.5 (2.4-6.7)	3.8 (2.4-5.1)	2.3 (1.2-3.5)	1.1 (-0.1-2.3)	1.2 (0.6-1.9)	<0.001	
Male	8.7 (6.8-10.6)	7.1 (5.1-9.0)	6.9 (4.9-8.9)	4.0 (2.3-5.8)	6.0 (3.9-8.1)	0.02	
Race							0.40
Non-Hispanic White	6.0 (4.6-7.3)	6.1 (4.1-8.1)	5.0 (2.6-7.2)	1.6 (0.04-3.2)	2.3 (0.8-3.8)	<0.001	
Non-Hispanic Black	9.1 (6.8-11.3)	5.9 (3.6-8.1)	7.0 (4.3-9.6)	4.7 (2.1-7.3)	6.8 (4.2-9.4)	0.18	
Hispanic	5.0 (3.4-6.6)	3.4 (1.8-5.1)	3.1 (2.0-4.2)	2.8 (1.4-4.2)	5.8 (3.7-7.9)	0.66	

© 2021 Hardy ST et al. *JAMA Network Open*.

Non-Hispanic Asian	.	.	.	3.1 (0.6-5.5)	2.9 (0.0-6.0)	0.76	
Non-Hispanic other	11.3 (2.3-20.3)	2.6 (0.0-5.8)	0.8 (0.0-2.4)	4.4 (0.0-8.9)	3.1 (0.0-6.8)	0.09	
Body mass index							0.03
Normal	4.8 (3.2-6.4)	4.2 (2.5-5.9)	3.2 (2.1-4.2)	1.8 (1.1-2.4)	0.9 (0.3-1.6)	<0.001	
Overweight	4.6 (2.0-7.3)	7.1 (4.4-9.7)	6.1 (0.9-11.3)	1.9 (0.4-3.4)	4.6 (1.5-7.7)	0.41	
Obese	17.2 (13.1-21.2)	9.0 (5.8-12.9)	9.0 (4.5-13.4)	5.3 (2.0-8.6)	10.8 (7.3-14.3)	0.03	
Poverty-to-income ratio							0.04
<1.30	5.5 (3.9-7.1)	3.5 (2.1-4.9)	3.7 (1.6-5.7)	3.2 (1.9-4.4)	3.3 (1.3-5.3)	0.10	
1.30–3.49	6.3 (4.1-8.5)	6.1 (3.0-8.4)	5.2 (3.0-7.4)	1.6 (0.7-2.4)	5.5 (3.2-7.8)	0.10	
≥3.50	7.3 (5.0-9.6)	5.8 (3.9-7.6)	4.2 (1.9-6.4)	3.2 (0.8-5.6)	1.2 (0.2-2.2)	<0.001	

NHANES: National Health and Nutrition Examination Survey, SE: standard error

[*]P value for trend in age-adjusted blood pressure levels across all survey years.

[†] Non-Hispanic Asians were not presented in 1999-2002, 2003-2006, and 2007-2010 due to small sample sizes.

[‡] Body mass index was categorized as normal (body mass index 5th percentile to < 85th percentile), overweight (body mass index 85th percentile to < 95th percentile) and obese (body mass index ≥ 95th percentile) based on age- and sex-specific growth charts developed by the Centers for Disease Control and Prevention in 2000. Due to a small sample size, children with a body mass index < 5th percentile are not presented.

[§]A poverty-to-income ratio < 1.30 indicates that the family income was below 130% of the poverty level.

Age-adjustment was performed using direct standardization with the standard being US children and adolescents across the entire time period from 1999 to 2018.

© 2021 Hardy ST et al. *JAMA Network Open*.

eFigure. Flowchart Showing the Number of NHANES Participants Included in the Current Analyses

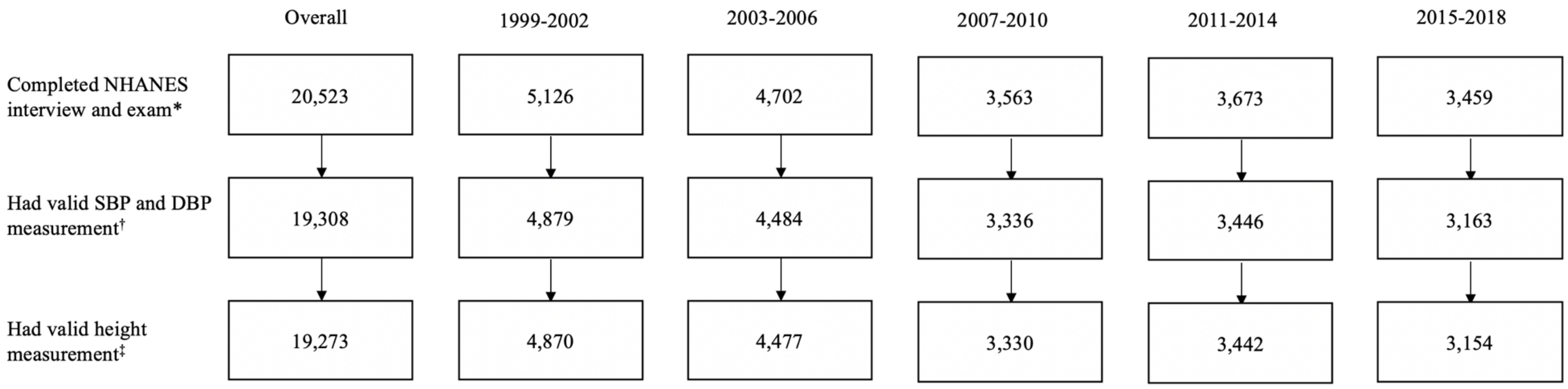

NHANES: National Health and Nutrition Examination Survey; SBP: systolic blood pressure; DBP: diastolic blood pressure.
* NHANES participants 8 to 17 years old who completed the interview and exam.
† Had at least one valid SBP and one DBP measurement.
‡ This criteria only applies to those 8-12 years of age. For this age group, height is used for the determination of blood pressure percentiles for defining elevated blood pressure and hypertension.

© 2021 Hardy ST et al. *JAMA Network Open*.
